# Coiling followed by staged flow diversion for large and giant intracranial aneurysms

**DOI:** 10.3389/fneur.2022.1024447

**Published:** 2022-12-01

**Authors:** Nan Lv, Hongyu Ma, Yu Zhou, Zhiqing Li, Yiyong Zeng, Qiang Li, Rui Zhao, Yibin Fang, Pengfei Yang, Qinghai Huang, Bo Hong, Yi Xu, Zhiqing Lin, Jianmin Liu

**Affiliations:** ^1^Neurovascular Center, Changhai Hospital, Naval Medical University, Shanghai, China; ^2^Shengjing Hospital of China Medical University, Shenyang, China; ^3^Ningbo City First Hospital, Ningbo, China

**Keywords:** intracranial aneurysm, flow diverter, complication, delayed aneurysm rupture, endovascular treatment

## Abstract

**Objective:**

Delayed aneurysm rupture is a fatal complication after flow diversion treatment for large and giant intracranial aneurysms. This study aimed to investigate the feasibility and safety of coiling first and followed by planned flow diversion to prevent delayed aneurysm rupture.

**Methods:**

From January 2017 to December 2021 in two institutions, patients with unruptured intracranial aneurysms treated by coiling first and planned flow diversion were retrospectively collected. Data on demographic and aneurysmal characteristics, procedural details, and clinical and angiographic outcomes were reviewed.

**Results:**

Thirty patients were included (7 Males and 23 Females; Median age 57 years). Aneurysmal size ranged from 11.8 to 26.8 mm, with a median value of 18.5 mm. All aneurysms were located within the intradural segment of internal carotid arteries. Coiling and planned flow diversion were successfully performed in all patients. The time interval between coiling and flow diversion was 3.9–6.7 weeks, with a median value of 5.2 weeks. No hemorrhagic or ischemic complications occurred during the procedures and follow-up. Complete or subtotal occlusion was achieved in 86.7% (26/30) at the last angiographic follow-up (median 6.7 months).

**Conclusion:**

The preliminary data suggested that coiling unruptured intracranial aneurysms followed by planned flow diversion is both safe and effective. Further studies with larger cohorts are needed to verify the effect of this new strategy in preventing delayed rupture after flow diversion.

## Introduction

Flow diverter (FD) has been an optimal endovascular treatment technique for large and giant intracranial aneurysms (IAs), and its indication continues to be expanded (1). Despite the high occlusion rate, complications of FD treatment could not be ignored. Delayed aneurysm rupture is a rare but fatal complication after FD placement (2, 3). The mechanism of this complication has not been well established, but it is believed that the interaction between hemodynamic changes caused by FD and sequential thrombosis formation plays a crucial role. Some hemodynamic studies revealed that FD placement could result in dramatic and undesirable flow changes, which increased intra-aneurysmal pressure and wall tension (4).

Concomitant coiling with FD is suggested by some authors to prevent delayed aneurysm rupture by obstructing inject flow (fast and concentrated contrast agent flow shooting from aneurysm neck to aneurysm sac on 2D-DSA) and accelerating thrombosis formation (5). However, there is no reliable evidences that concomitant coiling could eliminate delayed rupture. As shown in the review of Rouchaud et al. (3), 20 % of aneurysms that experienced delayed rupture were coiled. This could be explained by that though concomitant coiling could accelerate the thrombosis process, there's still a time lag before stable aneurysm obliteration by thrombosis.

Based on the existing knowledge of the mechanism of delayed rupture, we hypothesize that coiling first to induce adequate intra-aneurysmal thrombosis formation and then implanting FD in a second stage would benefit to reduce the risk. Here, we reported a cohort of unruptured intracranial aneurysms that were treated with this preliminary modality to investigate its feasibility and safety.

## Methods

The Institution Review Boards of Changhai Hospital and Ningbo City First Hospital approved this retrospective study. All the patients were informed of staged coiling followed by flow diversion treatment for their intracranial aneurysms.

### Patients selection

Between January 2017 and December 2021 in the two institutions, 30 patients with unruptured intracranial aneurysms were treated with coiling followed by planned FD implantation. The demographic characteristics, clinical presentation, aneurysmal morphology, and location were collected for the 30 cases (Table 1).

**Table 1:** Characteristics of patients and aneurysms.

**Characteristic**	**Value**
**Gender [*****n*** **(%)]**	
Male	7 (23.3)
Female	23 (76.7)
**Age (years)**	
Range	25–68
Median (IQR)	57 (48, 61)
Hypertension [*n* (%)]	7 (23.3)
Diabetes [*n* (%)]	5(16.7)
Smoking [*n* (%)]	8 (26.7)
Family history of aSAH [*n* (%)]	1 (3.3)
**Clinical presentation [*****n*** **(%)]**	
Headache	10 (33.3)
Visual symptoms	7 (23.3)
Incidentally	7 (23.3)
Dizziness	5 (16.7)
Ischemic events	1 (3.3)
**Aneurysm location [*****n*** **(%)]**	
Ophthalmic Segment	13 (43.3)
Supraclinoid Segment	13 (43.3)
Communicating Segment	4 (13.3)
**Maximum diameter (mm)**	
Range	11.8–26.8
Median (IQR)	18.5 (16.1, 20.9)
**Neck diameter (mm)**	
Range	2.9–9.0
Median (IQR)	5.9 (5.3, 6.5)
**Dome to neck ratio**	
Range	2.0–4.9
Median (IQR)	3.1 (2.9, 3.5)

Staged coiling followed by flow diversion treatment would be conducted when an aneurysm met the following characteristics: (1) maximum diameter > 10 mm (large, 10–25 mm; giant, >25 mm); (2) relatively narrow neck (dome to neck ratio ≥2.0); (3) inject flow sign; (4) located within the intradural segment of cerebral arteries.

### Endovascular procedures and antiplatelet protocol

All endovascular procedures were performed under general anesthesia and systematic heparin. *Via* femoral artery approach, a femoral artery sheath and a guiding catheter were used to provide proximal support. In the first stage of coiling, microcatheters were pre-shaped and guided by microwires into aneurysm sacs to deliver coils. The staged FD was performed at least 4 weeks after coiling to allow adequate thrombosis formation within the aneurysm. During the interval period, phone call follow-up was conducted to confirm the safety of patients. They were advised to accept staged FD within one month after the delay for thrombosis formation. After FD implant, 80 mg methylprednisolone was given intravenously to prevent the risk of rupture associated with thrombus-induced inflammation. FDs used in our institutions were Tubridge Flow Diverter (TFD, MicroPort, Shanghai, China) and Pipeline Embolization Device (PED, Medtronic, USA). Both TFDs and PEDs were developed through a dedicated microcatheter (for TFD: T-track, MicroPort; for PED: Marksman, Medtronic) using the push-pull technique. In cases that required further coiling, another microcatheter would be positioned in the aneurysm sac before the deployment of FDs.

Each patient received dual antiplatelet treatment (100 mg/day aspirin plus 75 mg/day clopidogrel) for at least 3 days before FD implantation. A postoperative antiplatelet regimen was administered as follows: <3 months: 100 mg aspirin+75 mg clopidogrel; ≥3 months: 100 mg aspirin indefinitely. For patients with clopidogrel resistance according to Thrombelastography (TEG) examination, ticagrelor 90 mg twice a day would be given.

### Angiographic outcomes evaluation

All patients were advised to undergo angiographic follow-up 6 months after the treatment and annually thereafter. Two experienced neuroradiologists interpreted the angiography images together to avoid bias. Angiographic outcomes of coiling and FD treatment were classified into 3 categories to allow comparison between different treatment phases: (a) complete occlusion, no contrast filling of the aneurysm sac; (b) subtotal occlusion, minor residual sac filling or neck remnant; (c) incomplete occlusion, substantial residual sac filling.

## Results

### Patients and aneurysms

There were 23 (76.7%) women and 7 (23.3%) men, with a median age of 57 years (range 25–68 years). Aneurysms were detected for headache in 10 (33.3%) patients, visual symptoms in 7 (23.3%) patients, dizziness in 5 (16.7%) patients, transient ischemic stroke in 1 (3.3%) patient, and incidentally found in 7 (23.3%) patients.

Of the 30 aneurysms, 2 were Giant aneurysms (>25 mm) and the others were large aneurysms (10–25 mm). All the cases were saccular aneurysms. The maximum diameters of the intracranial aneurysms ranged from 11.8 to 26.8 mm, with a median size of 18.5 mm (IQR 16.1–20.9 mm). The aneurysm neck ranged from 2.9 to 9.0 mm, with a median value of 5.9 mm (IQR 5.3–6.5 mm). The dome-to-neck ratio (maximum diameter/neck width) of the aneurysms ranged from 2.0 to 4.9, with a median value of 3.1 (IQR 2.9–3.5). All aneurysms were located at intradural segments of internal carotid arteries, with 13 (43.3%) aneurysms at the ophthalmic segment, 13 (43.3%) at the supraclinoid segment, and 4 (13.3%) at the communicating segment.

### Clinical outcomes

All procedures, coiling and staged FD implantation, were successfully performed. The treatment characteristics of all patients were listed in Table 2. TFDs were implanted in 21 aneurysms and PEDs in 9 aneurysms. Balloon remodeling was needed in 2 (6.7%) patients, in which the position of FD was not satisfied. Additional coiling was performed in 3 (10.0%) patients during the staged procedures. No procedure-related hemorrhagic or ischemic events occurred. Clinical follow-up ranged from 4.4 to 15.3 months, with a median interval of 6.7 months (IQR 5.8–8.6 months). No deterioration of the mRS score was observed during follow-up. Of the 7 patients with vision deficits, improvement of vision happened in 2 patients and the others were stable.

**Table 2:** Treatment characteristics of the patients.

**Characteristic**	**Value**
**Interval between Coiling and FD (weeks)**	
Range	3.9–6.7
Median (IQR)	5.2 (4.3, 6.0)
**FD Type [*****n*** **(%)]**	
Tubridge	21 (70.0)
Pipeline	9 (30.0)
**Adjunctive Techniques with FD [*****n*** **(%)]**	
Balloon remodeling	2 (6.7)
Further coiling	3 (10.0)
**Procedural-related complication [*****n*** **(%)]**	
Hemorrhagic complication	0 (0.0)
Ischemic complication	0 (0.0)
**Duration of Follow-up after FD (months)**	
Range	4.4–15.3
Median (IQR)	6.7 (5.8, 8.6)

FD, flow diverter.

### Angiographic outcomes

**Figure 1:**
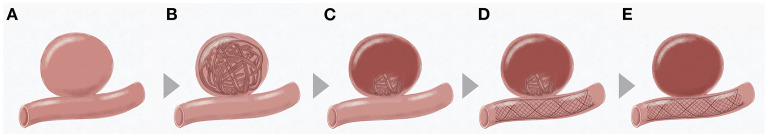
Flow chart of coiling followed by staged flow diversion for unruptured intracranial aneurysms. **(A)** Sketch of an intracranial aneurysm; **(B)** First-stage coiling; **(C)** Thrombosis formation induced by coiling; **(D)** Second-stage flow diversion; **(E)** Complete occlusion after flow diversion.

Angiographic outcomes in all treatment stages were shown in Table 3. The obliteration process of the aneurysm was illustrated in Figure 1. After first-stage coiling, the immediate results showed that complete occlusion was achieved in 1(3.3%) patient, subtotal occlusion in 5 (16.7%), and incomplete occlusion in 24 (80.0 %).

**Table 3:** Radiologic outcomes.

	**No. of aneurysms (%)**			
**Angiographic outcomes**	**Immediate after coiling**	**Pre-flow diverter**	**Immediate after flow diverter**	**Last follow-up**
Complete occlusion	1 (3.3)	1 (3.3)	1 (3.3)	19 (63.3)
Subtotal occlusion	5 (16.7)	4 (13.3)	7 (23.3)	7 (23.3)
Incomplete occlusion	24 (80.0)	25 (83.3)	22 (73.3)	4 (13.3)

The median delay of staged FD implantation was 5.2 weeks (range 3.9–6.7 weeks; IQR 4.3–6.0 weeks). The angiography right before FD implantation showed complete occlusion in 1(3.3%) patient, subtotal occlusion in 4 (13.3%), and incomplete occlusion in 25 (83.3%). Compared to the immediate results of initial coiling, coil compression was observed in 9 (30.0%) aneurysms, improvement in 14 (46.7%), and unchanged in 7 (23.3%). Immediately after FD implantation, the degree of occlusion was improved in 10 aneurysms (3 from incomplete to subtotal occlusion; 7 remained incomplete but with a reduction of filling volume).

**Figure 2:**
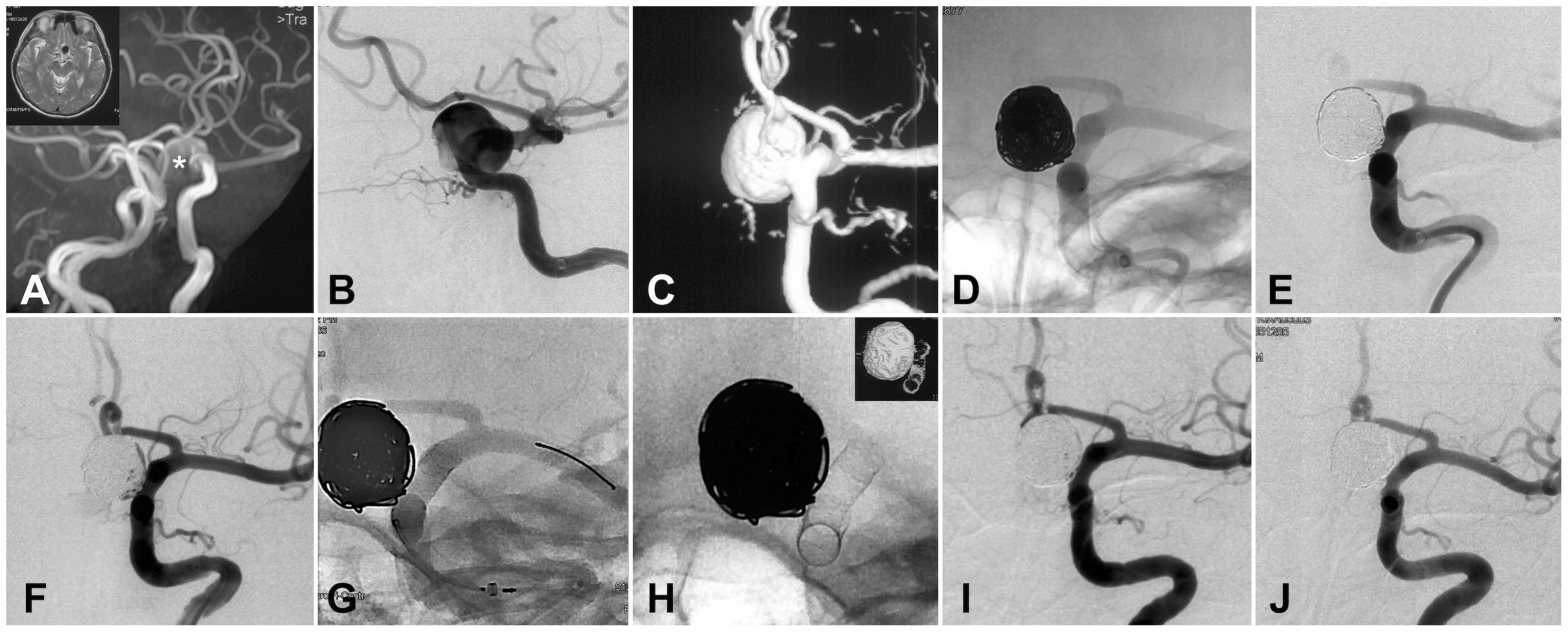
Illustration of coiling and staged flow diverter treatment for a large intracranial aneurysm. **(A)** CT and CT angiography identified the giant internal carotid aneurysm. **(B)** DSA confirmed the size and location of the aneurysm. **(C)** Three-dimension re-construction of the DSA. **(D)** First-stage coiling of the giant aneurysm. **(E)** Angiography after first-stage coiling. **(F)** DSA image right before the staged flow diverter (FD) treatment. **(G)** The procedure of FD implantation. **(H)** The shape and location of the implanted FD. **(I)** Angiography immediately after FD implantation. **(J)** Six-month follow-up DSA showed the complete occlusion of the giant aneurysm. *The position of aneurysm cavity.

The duration of follow-up after FD treatment ranged from 4.4 to 15.3 months, with a median value of 6.7 months (IQR 5.8–8.6 months). At the last follow-up, complete occlusion was achieved in 19 (63.3%) patients, subtotal occlusion in 7 (23.3%) patients, and incomplete occlusion in 4 (13.3%) patients. No in-stent stenosis was observed during the follow-up. All covered branches (17 ophthalmic arteries, 8 anterior choroid arteries, and 5 posterior communicating arteries) were patent, except for 2 cases where the flow of covered ophthalmic arteries reduced without symptom. Figure 2 displayed the treatment procedures and 6-month follow-up outcome in an aneurysm located at the ophthalmic segment. The example cases for aneurysms located at the supraclinoid segment and the communicating segment were attached at Supplementary Figures S1, S2.

## Discussion

Delayed aneurysm rupture is a serious complication after FD treatment with extremely high mortality (6). According to a current review, the overall incidence of delayed aneurysm rupture after FD treatment is 1.8% (2). In this study, we proposed a preliminary modality that coiling followed by staged FD for unruptured large and giant IAs. No procedure-related complication occurred and the angiographic outcomes were relatively satisfied, which might suggest the feasibility and safety of this staged strategy.

The concept of coiling followed by staged flow diversion has been introduced in acute ruptured intracranial aneurysms previously. Brinjikji et al. (7) reported 31 patients with complex ruptured intracranial aneurysms by acute coiling followed by staged flow diversion and concluded that the strategy is both safe and effective. Howard et al. (8) also reported a cohort of 22 patients with ruptured intracranial aneurysms treated with staged flow diversion and achieved a subtotal occlusion rate of 91% in follow-up. For unruptured large and giant aneurysms, this strategy could also be effective because thrombosis formation is equally important. The delayed ruptured cases resulted from sudden but undesirable flow changes, such as increasing intra-aneurysmal pressure and aneurysm wall tension (4, 9, 10).

Previously, various attempts have been made to prevent delayed aneurysm rupture by enhancing the flow diversion effect and accelerating thrombosis formation. Concomitant coiling with FD is suggested to help accelerate thrombosis formation (5, 11, 12). Park et al. (12) reported that concomitant coiling with FD resulted in a significantly lower retreat rate compared with FD alone. Similarly, Bender et al. (5) shared their experience of single-stage FD with coiling and concluded that coiling can expedite and improve occlusion outcomes without a significant increase in morbidity. Despite these desirable results, the effect of concomitant coiling on preventing delayed rupture is not clear. Indeed, according to a literature review by Rouchaud et al. (3), over 80% of aneurysms that ruptured after FD treatment were not previously coiled; but they also pointed out that coiling might not be a panacea as 20% of delayed rupture happens in aneurysms with concomitant coiling. Although coiling has shortened the process of thrombosis formation, there is still a time lag before adequate thrombosis is established.

Therefore, we hypothesized that coiling first, which can accelerate the process of stable thrombosis formation by occupied effect and constantly disturbing the blood flow perfused into the aneurysm sac, and then implanting FD in a second stage would help to reduce the rupture risk caused by those harmful flow changes. In theory, the thrombosis induced by first-stage coiling could provide more protection for the aneurysmal dome by resisting those dramatic and harmful flow changes caused by staged FD placement The intra-aneurysmal pressure increases would be milder so the possible rupture of an aneurysm might be reduced. In our series, no hemorrhagic complication occurred in all 30 cases, either in the interval between initial coiling and staged FD placement, or after FD placement, which might suggest the feasibility and safety of staged FD for selected lesions. Another priority is that the staged strategy could avoid the usage of anti-platelet medicine in the acute phase, which would favor thrombosis formation inside aneurysms. In addition, the staged FD treatment makes the technique much easier and safer than the single-stage contaminant coiling with FD. In particular, the staged modality would shorten the single procedural time and reduce technical complexity. After FD placement, 80 mg methylprednisolone would be continuously used for 3 days to prevent the possible rupture resulting from thrombus-induced inflammation.

Despite the possible benefit of staged-FD treatment, several concerns need to be noticed. Firstly, patient selection for staged FD is driven by various factors. The compliance with dual antiplatelet therapy and imaging follow-up is most important for selecting patients. The staged strategy should not be recommended to a potentially noncompliant patient. Secondly, second-stage FD placement should be applied timely to avoid significant recurrence after coiling. In the present series, the interval staged FD placement after coiling ranged from 3.9 to 6.7 weeks, with a median value of 5.2 weeks. During this interval, 9 aneurysms presented with acceptable coil compression before staged FD placement. Lastly, this treatment is more costly compared with stent-assisted coiling or FD placement alone. The average cost for coiling plus staged-FD is more than 40,000 USD. Therefore, the risk of rupture and benefit from the operation needs to be fully evaluated before the decision for staged-FD treatment.

Based on the experience of delayed rupture from literature and our clinical practice, we proposed several factors that might result in high delayed rupture risk and should consider coiling first and followed by staged FD.

(1) Intra-dural lesions. The coiling and staged FD technique might be more meaningful for aneurysms distal to the dural ring, which may cause fatal intracranial hemorrhage. (2) Large and giant aneurysms. Several studies have suggested a higher risk of rupture, especially delayed rupture, in large and giant aneurysms (3, 13–15). (3) Aneurysm geometry. Several geometry parameters, for example, aspect ratio (AR) and dome-to-neck ratio (DNR), rely on the width of the neck. Aneurysms with a larger DNR had been proven to correlate with increasing rupture risk (16, 17). In addition, a relatively narrow neck could provide stability for coils, making coiling easy and safe. (4) Flow pattern. Previous hemodynamic studies suggested that the impinging inflow is associated with increased tension of the aneurysm wall and increased rupture risk (18). In a review of 13 cases of delayed rupture aneurysms by Kulcsar et al. (14), an inflow jet was observed in all cases on DSA images. Coiling and the consequent thrombosis could help to eliminate the direct impact of the inflow jet on the aneurysm wall, and this effect could be enhanced by the staged FD implantation.

This study has several limitations. Firstly, the retrospective design might add obvious bias to patient selection. Secondly, while no delayed aneurysm rupture occurred in this cohort, these data might not be generalizable considering the small sample size. Further studies, both clinical observations, and animal experiments are warranted to determine the mechanism, indication, appropriate interval, and other technical details. Also, no posterior circulation aneurysms were included in this cohort due to the small sample size. Thirdly, some patients enrolled in this study could otherwise be treated by other strategies such as surgical clipping and stent-assisted coiling. The comparison among different strategies was not made in this study. Lastly, the follow-up periods were short. There were incompletely occluded aneurysms at the last follow-up. Long-term follow-up is manipulated to investigate the efficacy and durability of this treatment. Lastly, the cost-effectiveness of this new strategy should also be further discussed.

## Conclusion

Overall, our data suggested that coiling followed by planned flow diversion is both safe and effective for unruptured intracranial aneurysms with a potentially high risk of delayed rupture. Further studies with prospective design and increasing sample size are warranted to determine the effect of this staged modality on preventing delayed aneurysm rupture.

## Data availability statement

The original contributions presented in the study are included in the article/Supplementary material, further inquiries can be directed to the corresponding authors.

## Ethics statement

The studies involving human participants were reviewed and approved by the Ethics Committee of Changhai Hospital. The patients/participants provided their written informed consent to participate in this study.

## Author contributions

JL and ZL: conception or design of the work. YZh, ZL, and YZe: acquisition of data. QL, RZ, and YF: analysis of data. PY and QH: interpretation of data. NL and HM: drafting the work. BH, YX, and ZL: revising the work. JL: final approval of the version. All authors contributed to the article and approved the submitted version.

## Funding

This work was supported by National Research and Development Project of Key Chronic Diseases (Grant No. 2016YFC1300700), National Natural Science Foundation of China (Grant No. 81701775), the Project of Shanghai Municipal Health Commission (Grant No. 20194Y0131), and the Project from Shanghai Science and Technology Commission (Grant No. 19DZ1930302).

## Conflict of interest

The authors declare that the research was conducted in the absence of any commercial or financial relationships that could be construed as a potential conflict of interest.

## Publisher's note

All claims expressed in this article are solely those of the authors and do not necessarily represent those of their affiliated organizations, or those of the publisher, the editors and the reviewers. Any product that may be evaluated in this article, or claim that may be made by its manufacturer, is not guaranteed or endorsed by the publisher.

## Supplementary material

Supplementary Figure S1 Illustration of coiling and staged flow diverter treatment for a large intracranial aneurysm located at supraclinoid segment. **(A**) The anteroposterior angiography identified size and location of the giant internal carotid aneurysm. **(B)** The lateral angiography confirmed morphology the aneurysm. **(C)** Three-dimension re-construction of the DSA. **(D)** First-stage coiling of the giant aneurysm. **(E)** Angiography after first-stage coiling. **(F)** DSA image right before the staged flow diverter (FD) treatment. **(G)** The shape and location of the implanted FD. **(H)** The parent artery was unaffected after the implanted FD. **(I)** Angiography immediately after FD implantation. **(J)** Six-month follow-up DSA showed the complete occlusion of the giant aneurysm.

Supplementary Figure S2 Illustration of coiling and staged flow diverter treatment for a large intracranial aneurysm located at communicating segment. **(A)** The anteroposterior angiography identified the giant internal carotid aneurysm. **(B)** The lateral angiography confirmed location and size of the aneurysm. **(C)** Three-dimension re-construction of the DSA. **(D)** First-stage coiling of the giant aneurysm. **(E)** Angiography after first-stage coiling. **(F)** DSA image right before the staged flow diverter (FD) treatment. **(G)** The procedure of FD implantation. **(H)** The shape and location of the implanted FD. **(I)** Angiography immediately after FD implantation. **(J)** Six-month follow-up DSA showed the complete occlusion of the giant aneurysm.
