# Supplementary figures and images for: Coiling followed by staged flow diversion for large and giant intracranial aneurysms
Source: Front Neurol. 2022 Dec 1;13:1024447. doi: 10.3389/fneur.2022.1024447 (PMC9751354; doi:10.3389/fneur.2022.1024447)

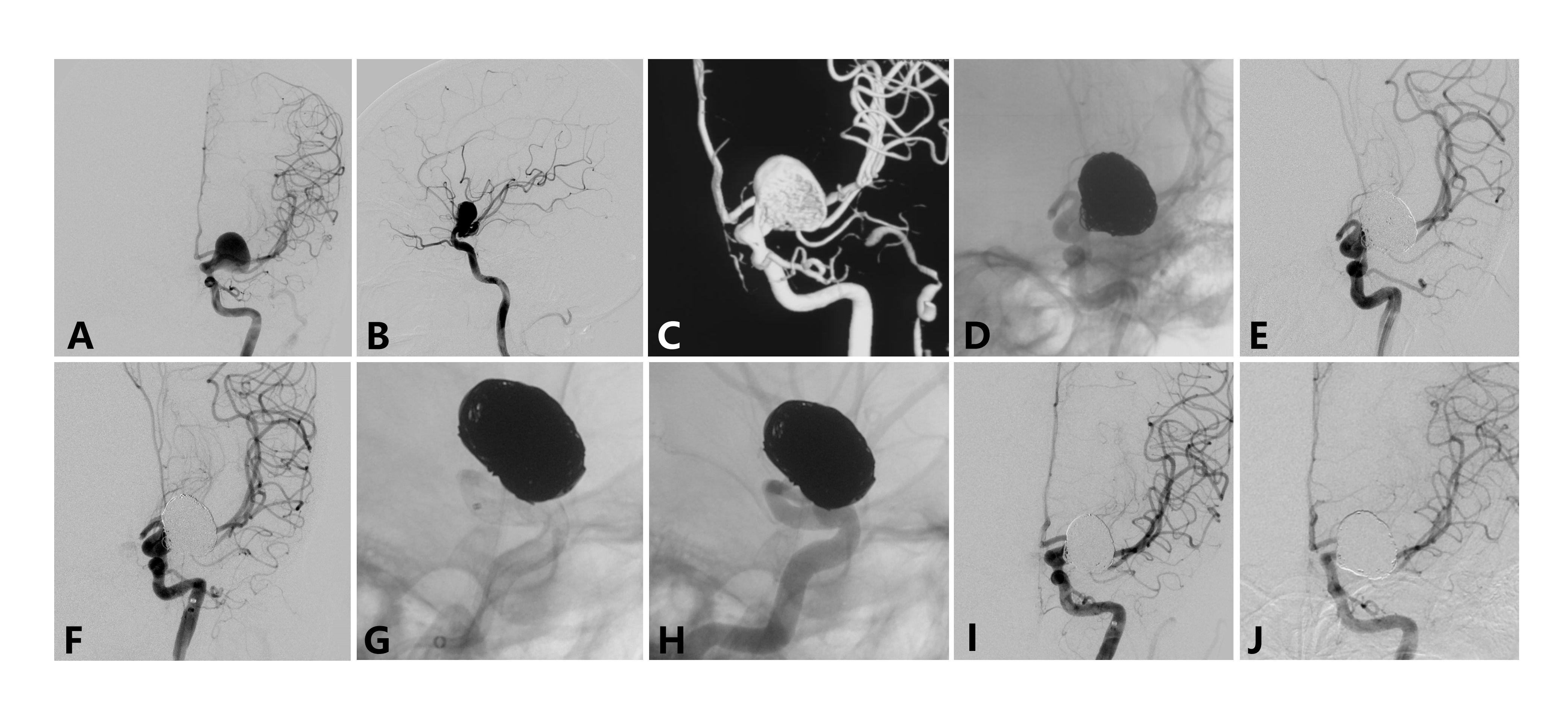

Supplement: Supplementary Figure S1 — Illustration of coiling and staged flow diverter treatment for a large intracranial aneurysm located at supraclinoid segment. (A) The anteroposterior angiography identified size and location of the giant internal carotid aneurysm. (B) The lateral angiography confirmed morphology the aneurysm. (C) Three-dimension re-construction of the DSA. (D) First-stage coiling of the giant aneurysm. (E) Angiography after first-stage coiling. (F) DSA image right before the staged flow diverter (FD) treatment. (G) The shape and location of the implanted FD. (H) The parent artery was unaffected after the implanted FD. (I) Angiography immediately after FD implantation. (J) Six-month follow-up DSA showed the complete occlusion of the giant aneurysm. [file Image_1.PNG]

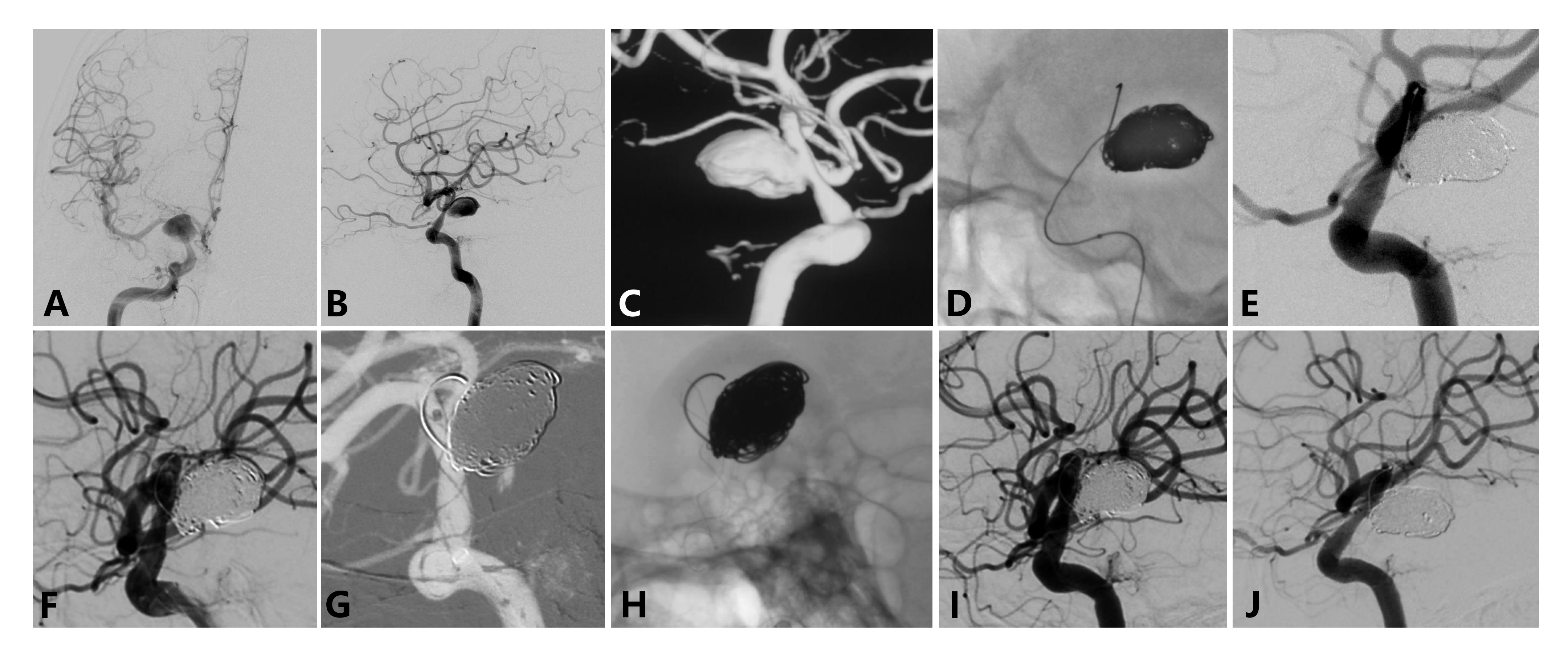

Supplement: Supplementary Figure S2 — Illustration of coiling and staged flow diverter treatment for a large intracranial aneurysm located at communicating segment. (A) The anteroposterior angiography identified the giant internal carotid aneurysm. (B) The lateral angiography confirmed location and size of the aneurysm. (C) Three-dimension re-construction of the DSA. (D) First-stage coiling of the giant aneurysm. (E) Angiography after first-stage coiling. (F) DSA image right before the staged flow diverter (FD) treatment. (G) The procedure of FD implantation. (H) The shape and location of the implanted FD. (I) Angiography immediately after FD implantation. (J) Six-month follow-up DSA showed the complete occlusion of the giant aneurysm. [file Image_2.PNG]
